# Decoding iNOS Inhibition: A Computational Voyage of Tavaborole Toward Restoring Endothelial Homeostasis in Venous Leg Ulcers

**DOI:** 10.3390/ph19010137

**Published:** 2026-01-13

**Authors:** Naveen Kumar Velayutham, Chitra Vellapandian, Himanshu Paliwal, Suhaskumar Patel, Bhupendra G. Prajapati

**Affiliations:** 1 Department of Pharmacology, Faculty of Medicine and Health Sciences, SRM Institute of Science and Technology, SRM College of Pharmacy, Kattankulathur, Chengalpattu 603 203, Tamil Nadu, India; nv1281@srmist.edu.in; 2 Marwadi University Research Center, Faculty of Pharmacy, Marwadi University, Rajkot 360003, Gujarat, India; himanshu.paliwal@marwadieducation.edu.in; 3 Formulation Research and Development, Amneal Pharmaceutical, Piscataway, NJ 08854, USA; suhas185@gmail.com; 4 Centre for Research Impact & Outcome, Chitkara College of Pharmacy, Chitkara University, Rajpura 140401, Punjab, India; 5 Department of Pharmaceutics, Parul Institute of Pharmacy, Faculty of Pharmacy, Parul University, Waghodia, Vadodara 391760, Gujarat, India

**Keywords:** iNOS inhibition, tavaborole, venous leg ulcer, molecular docking, molecular dynamics, MM-PBSA, nitrosative stress, wound healing

## Abstract

**Background:** Due to chronic venous insufficiency, venous leg ulcers (VLUs) develop as chronic wounds characterized by impaired healing, persistent inflammation, and endothelial dysfunction. Nitrosative stress, mitochondrial damage, and tissue apoptosis caused by excess nitric oxide (NO) produced by iNOS in macrophages and fibroblasts are contributing factors in the chronic wound environment; therefore, pharmacological modulation of iNOS presents an attractive mechanistic target in chronic wound pathophysiology. **Methods:** Herein, we present the use of a structure-based computational strategy to assess the inhibition of tavaborole, a boron-based antifungal agent, against iNOS using human iNOS crystal structure (PDB ID: iNOS) by molecular docking using AutoDock 4.2, 500 ns simulation of molecular dynamics (MD), with equilibration within ~50 ns and analyses over full trajectory and binding free energy calculations through the MM-PBSA approach. **Results:** Docking studies showed favorable binding of tavaborole (–6.1 kcal/mol) in the catalytic domain, which stabilizes contacts with several key residues (CYS200, PRO350, PHE369, GLY371, TRP372, TYR373, and GLU377). MD trajectories for 1 ns showed stable structural configurations with negligible deviations (RMSD ≈ 0.44 ± 0.10 nm) and hydrogen bonding, and MM-PBSA analysis confirmed energetically favorable complex formation (ΔG_binding ≈ 18.38 ± 63.24 kJ/mol) similar to the control systems (L-arginine and 1400W). **Conclusions:** Taken together, these computational findings indicate that tavaborole can stably occupy the iNOS active site and interact with key catalytic residues, providing a mechanistic basis for further in vitro and ex vivo validation of its potential as an iNOS inhibitor to reduce nitrosative stress and restore endothelial homeostasis in venous leg ulcers, rather than direct therapeutic proof.

## 1. Introduction

Venous leg ulcers (VLUs) are among the most prevalent and treatment-resistant forms of chronic wounds, contributing to nearly 70% of ulcerations in the lower extremities and affecting approximately 1% of the global adult population [1,2]. The condition mainly arises as a consequence of chronic venous insufficiency (CVI), marked by malfunctioning venous valves, venous reflux, and persistent hypertension that collectively result in microcirculatory dysfunction and long-term inflammation [3,4]. The sustained elevation of venous pressure damages the endothelium, enhances leukocyte adhesion, and activates inflammatory pathways, forming a self-perpetuating loop of oxidative injury, tissue hypoxia, and delayed repair. Although a range of management strategies—including compression therapy, pharmacological treatment, and surgical correction—are available, recurrence remains common and troubling, with reported rates reaching 50–70% within five years of initial healing. The complex interplay of hemodynamic, biochemical, and cellular factors underscores the necessity of identifying specific molecular targets that can resolve chronic inflammation and endothelial dysfunction while promoting repair [1,3].

In addition to competing with arginase-1 for the common substrate L-arginine, inducible nitric oxide synthase (iNOS) catalyzes the production of excessive nitric oxide (NO), which can disrupt the metabolic homeostasis needed for macrophage regulation. The arginase-1 and iNOS functional interplay is a crucial metabolic checkpoint that orchestrates macrophage polarization and influences the overall dynamics of wound healing [5,6]. While arginase-1 activity promotes the reparative M2 macrophage phenotype involved in extracellular matrix synthesis and tissue remodeling, increased iNOS activity promotes the inflammatory M1 phenotype that prolongs tissue damage and delays recovery [7]. Consequently, dysregulation of L-arginine metabolism is one of the primary causes of chronic wound persistence. Pharmacological modulation of iNOS holds promise for restoring redox balance and moving wounds from the inflammatory to the proliferative phase; experimental inhibition of iNOS has been associated with reduced oxidative damage, improved angiogenesis, and enhanced collagen deposition in animal wound models [8]. Selective inhibitors such as aminoguanidine and 1400W are potent anti-inflammatory and cytoprotective agents under conditions of ischemia, diabetic ulcers, and arthritis [9,10]. However, systemic toxicity, poor pharmacokinetics, and insufficient selectivity limit their clinical translation, underscoring the need for safer, topically compatible substitutes. Selective iNOS inhibition may also have antimicrobial and pro-angiogenic effects in venous ulcers, where microbial infection and inflammation coexist, stabilizing the extracellular matrix and fostering tissue healing. iNOS thus represents a mechanistically compelling upstream target in VLU pathophysiology, driving nitrosative stress, vascular dysfunction, and impaired healing [11,12,13,14,15,16,17]. While several molecular targets, including matrix metalloproteinases (MMPs), have been implicated in extracellular matrix degradation during chronic wound healing, these enzymes largely represent downstream effectors of tissue injury. In contrast, iNOS functions as a central upstream regulator of the oxidative–inflammatory cascade in venous leg ulcers. Pathological overexpression of iNOS results in sustained nitric oxide (NO) production, which rapidly reacts with superoxide to form peroxynitrite (ONOO^−^), a highly reactive oxidant that activates MMPs, induces fibroblast senescence, and promotes endothelial apoptosis. Therefore, targeting iNOS offers a mechanistic advantage in chronic wound healing by intercepting the inflammatory cascade at its source, thereby preventing downstream proteolytic activation and restoring a wound microenvironment conducive to effective tissue repair [12,13,14,15,16,18].

Molecular docking, MD simulations, and calculations of binding free energy through MM-PBSA analysis are used for quantitative analysis of ligand affinity, conformational stability, and thermodynamic feasibility [19]. The catalytic domain of iNOS contains a heme prosthetic group and a tetrahydrobiopterin (BH_4_) cofactor needed for catalysis. The active-site residues GLU371, TRP372, and TYR373 are involved in NO synthesis and interact with its substrate L-arginine. Therefore, targeting this region with small molecules is a logical way to modulate iNOS selectively without interfering with other NOS isoforms [20]. Numerous synthetic and natural molecules have recently been highlighted by computational studies. However, few candidates have been evaluated for topical application or in the context of venous wound pathology.

Drug repurposing exhibits a cost-effective route for identifying novel therapeutic uses for approved agents with established safety and pharmacokinetic profiles [21]. Tavaborole was selected based on our previous computational identification of its putative dual engagement with arginase-1 (prior work) and potential iNOS activity (current study), evaluated independently using structure-based approaches. Chemically, tavaborole is characterized by a benzoxaborole scaffold, in which the boron atom serves as a critical pharmacophore. This boron center functions as an electrophile capable of forming reversible covalent hemiboronate adducts with nucleophilic residues (such as serine or threonine hydroxyl groups) or activated water molecules within enzyme active sites. This unique “boron effect” enables high-affinity yet reversible binding, particularly to metalloenzymes and redox-regulated enzymes. Such a mechanism has been successfully exploited in editing-site inhibitors of leucyl-tRNA synthetase and provides a strong chemical rationale for exploring tavaborole as a modulator of L-arginine-metabolizing enzymes, including iNOS [22,23,24]. In our previous computational studies, we found that the boron-based antifungal agent tavaborole, which binds tightly to arginase-1 active-site residues, may be a candidate for repurposing as a therapy for venous ulcers [25]. In the current study, we extend the computational investigation to iNOS inhibition in chronic venous ulceration based on that framework. While iNOS suppression aims to decrease nitrosative stress, control excessive NO production, and recover endothelial homeostasis, arginase-1 inhibition promotes L-arginine for tissue repair. Conceptual dual modulation of arginase-1 and iNOS activities represents a hypothesis for reestablishing macrophage metabolic homeostasis, though simultaneous in vivo inhibition by tavaborole remains unproven and requires off-target toxicity and pathway-selectivity studies [26,27]. The inhibition of iNOS can break the inflammatory feedback loop, reduce the production of peroxynitrite, and inhibit endothelial apoptosis, making iNOS a target of clinical relevance in the treatment of venous ulcers because it connects vascular dysfunction, immune dysregulation, and oxidative injury [28,29]. Moreover, selective inhibition of iNOS may indirectly enhance endothelial nitric oxide synthase (eNOS)-mediated signaling, thereby supporting vasodilation and angiogenesis—key processes for tissue repair and regeneration [16]. A computational strategy involving structure-based approach, molecular docking, and long-term MD simulations can reveal the conformational dynamics of iNOS–ligand interactions, the binding specificity and energetics of compounds, and the stability, flexibility, and compactness of complexes, which can be thermodynamically validated by MM-PBSA calculations, which can support the rational optimization of potent iNOS inhibitor.

Therefore, the current study aims to find new small-molecule inhibitors of iNOS based on structure-guided molecular docking and 500 ns MD simulations with the human iNOS crystal structure (PDB ID: 3E7G), and to compare with the reference inhibitor 1400W to verify docking accuracy and free energy predictions. Ligand–protein interactions will be evaluated by root mean square deviation (RMSD), root mean square fluctuation (RMSF), radius of gyration (Rg), solvent-accessible surface area (SASA), hydrogen-bond dynamics, principal component analysis (PCA), and free energy landscape (FEL) profiling. MM-PBSA calculations will provide thermodynamic insights into binding stability and affinity to provide molecular basis for potential topical inhibitors of iNOS.

This study applies the computational workflow validated in our prior arginase-1 investigation to identify tavaborole as an iNOS-targeted candidate scaffold for venous leg ulcer therapy. These computational predictions suggest tavaborole may modulate nitrosative stress pathways, providing a molecular basis for future experimental validation and rational optimization of selective iNOS inhibitors rather than direct therapeutic proof. This framework establishes a computational foundation for exploring the redox–immune axis in chronic wound management.

## 2. Results

### 2.1. Molecular Docking

**Figure 1:**
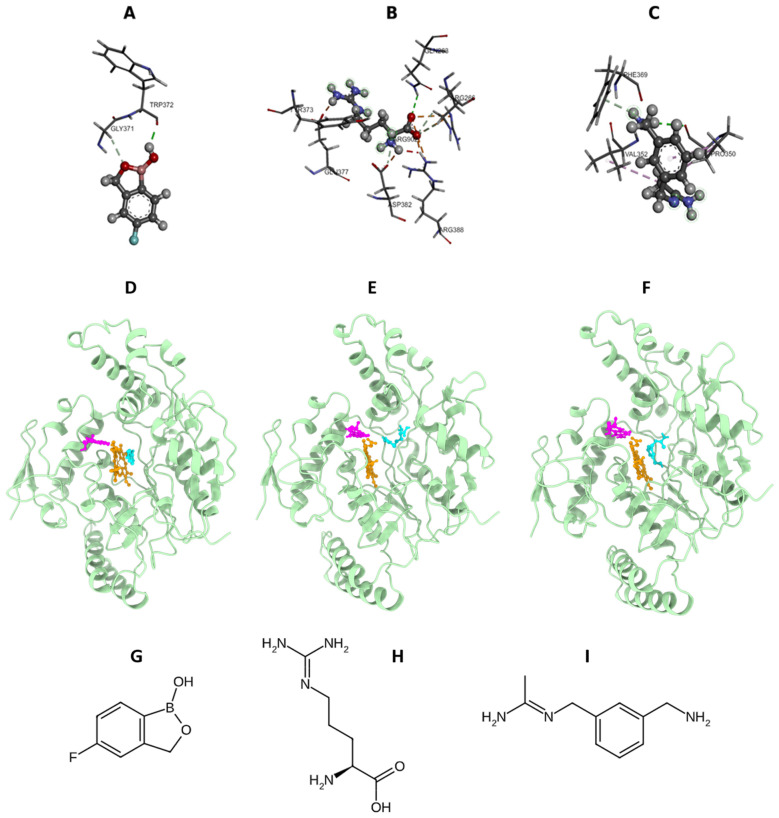
Molecular docking analysis of iNOS–ligand complexes. (**A**–**C**) Two-dimensional (2D) interaction maps illustrating key hydrogen bonding and hydrophobic interactions of (**A**) iNOS–Tavaborole (iNOS-TAV), (**B**) iNOS–L-arginine (iNOS-ARG), and (**C**) iNOS–1400W (iNOS-STD) within the catalytic active site of iNOS. Interacting residues are labeled to highlight critical binding determinants. (**D**–**F**) Three-dimensional (3D) docking poses of (**D**) iNOS-TAV, (**E**) iNOS-ARG, and (**F**) iNOS-STD, shown in ribbon representation of the iNOS protein with ligands displayed in ball-and-stick format. The heme cofactor (orange) and the co-crystallized tetrahydrobiopterin (BH_4_; magenta) are shown to delineate the catalytic pocket and ligand orientation. (**G**–**I**) Two-dimensional chemical structures of the ligands: (**G**) Tavaborole, (**H**) L-arginine, and (**I**) 1400W, provided for structural clarity and comparison.

The current molecular docking study showed that the protein–ligand complexes have different binding affinities based on their docking scores, which represent the strength of the molecular interactions, with the iNOS-Tavaborole complex having a docking score of –6.1, which indicates a favorable interaction with the target enzyme (Table 1). Tavaborole made contacts with several key residues in the catalytic domain: Cysteine 200, Proline 350, Alanine 351, Valine 352, Phenylalanine 369, Asparagine 370, Glycine 371, Tryptophan 372, Tyrosine 373, Methionine 374, and Glutamic acid 377, indicating that it is well oriented and stabilized in the active site. The broad range of interactions implies that Tavaborole fits well within the iNOS binding pocket, adopting a conformation that promotes strong intermolecular stability and contributes to its low docking energy. For comparison, the iNOS-Standard complex had a slightly better docking score of −6.8, indicating a stronger predicted affinity (Table 1). It binds to the same residues Glutamine 263, Tyrosine 347, Proline 350, Alanine 351, Valine 352, Phenylalanine 369, Asparagine 370, Glycine 371, Tryptophan 372, Tyrosine 373, and Glutamic acid 377, but the interaction is weaker, perhaps because of fewer interactions or a less ideal binding conformation (Figure 1A–C). The specific orientation of tavaborole within the active site is supported by π-π stacking with aromatic residues and hydrogen bonding with the catalytic triad. Although the docking scores of tavaborole (–6.1 kcal/mol) and L-arginine (–6.0 kcal/mol) are similar, tavaborole engages the catalytic pocket through extensive π-π stacking and hydrophobic contacts that are further characterized in the molecular dynamics and MM-PBSA analyses.

### 2.2. Molecular Dynamics

#### 2.2.1. Root Mean Square Deviation (RMSD)

**Figure 2:**
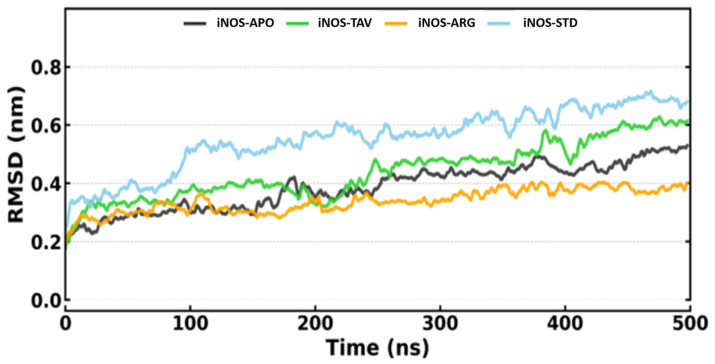
RMSD analysis of iNOS-APO, iNOS-TAV, iNOS-ARG, and iNOS-STD.

The values of RMSD were monitored over time (Figure 2), which indicated that all systems equilibrated by ~50 ns and remained stable throughout the 500 ns simulation with average RMSD values of 0.39 ± 0.08 nm (iNOS-APO, apo/ligand-free form), 0.44 ± 0.10 nm (iNOS-TAV, tavaborole-bound), 0.34 ± 0.04 nm (iNOS-ARG, L-arginine-bound), and 0.55 ± 0.11 nm (iNOS-STD, 1400W-bound), all within the accepted range (0.4–0.5 nm) for large, multi-domain enzymes like iNOS over long simulations. The iNOS-TAV complex plateaued early (~50 ns) with no substantial conformational drift relative to the apo protein or standard complexes. Consistent with the RMSD trends, the radius of gyration (Rg) profiles showed minimal variation across apo and ligand-bound systems, indicating preserved global compactness of iNOS throughout the simulation (Supplementary Figure S1).

#### 2.2.2. Root Mean Square Fluctuation (RMSF)

**Figure 3:**
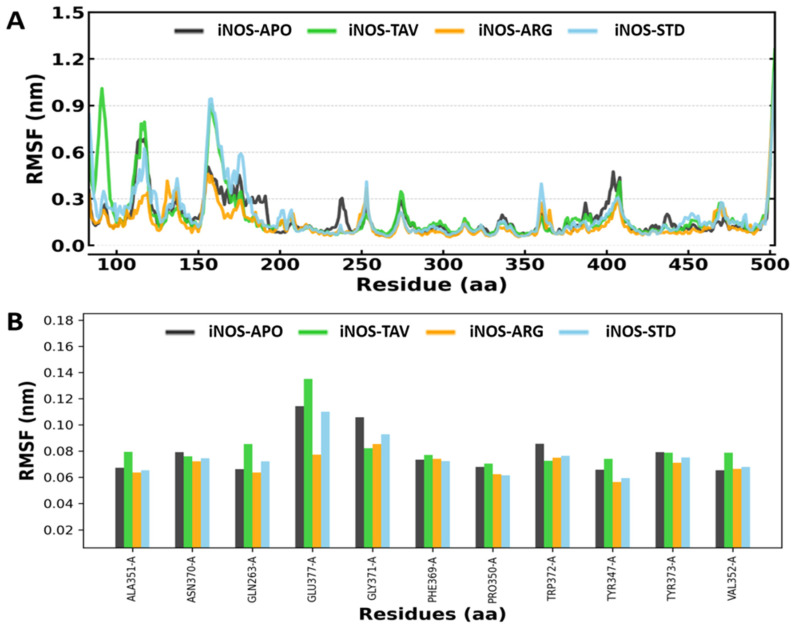
(**A**) RMSF analysis of iNOS-APO, iNOS-TAV, iNOS-ARG, and iNOS-STD (**B**) RMSF profiling of iNOS (iNOS) residues in the apo form and in complex with TAV, ARG and STD during 500 ns molecular dynamics simulation.

Typically, RMSFs are lower for compact structure of protein (such as helices and sheets) and higher for loosely ordered loop regions. The calculated values of RMSF for each complex for each residue in the iNOS-APO, iNOS-TAV, iNOS-ARG, and iNOS-STD complexes are depicted in Figure 3A,B, and the average RMSF value for iNOS-APO, iNOS-TAV, iNOS-ARG, and iNOS-STD was found to be 0.18 ± 0.13, 0.19 ± 0.17, 0.14 ± 0.10, and 0.19 ± 0.16 nm, respectively. Overall, the RMSF profiles of the iNOS-APO, iNOS-TAV, iNOS-ARG, and iNOS-STD systems showed comparable fluctuation patterns across most residues, indicating preserved global structural stability. Notably, the iNOS-TAV complex exhibited localized increases in RMSF around residues 90, 115, and 160. These regions correspond to solvent-exposed loop segments distal from the catalytic pocket and are not directly involved in substrate or cofactor binding. Such localized flexibility likely reflects ligand-induced adaptive conformational adjustments rather than global destabilization. Importantly, residues forming the iNOS catalytic site and ligand-binding region exhibited low RMSF values, supporting the structural stability of the iNOS–tavaborole complex throughout the simulation. RMSF analysis showed little fluctuation in flexibility in loop regions around the binding pocket, suggesting that ligand accommodation does not cause structural strain.

#### 2.2.3. Solvent Accessible Surface Area (SASA)

**Figure 4:**
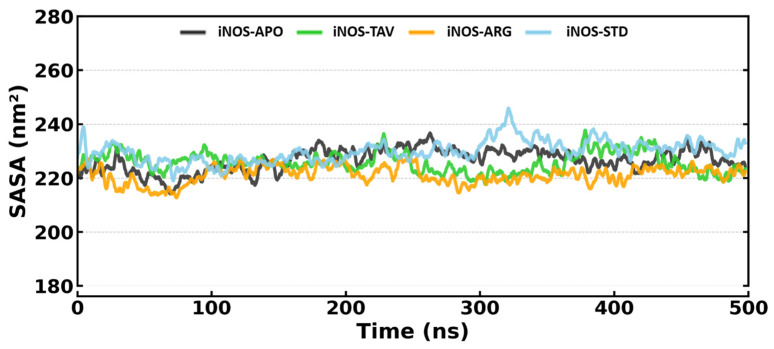
SASA analysis of iNOS-APO, iNOS-TAV, iNOS-ARG, and iNOS-STD.

The calculation of SASA values for iNOS-APO, iNOS-TAV, iNOS-ARG, and iNOS-STD were done and plotted (Figure 4) to see how binding affects the solvent accessibility of the target. The plot shows that the SASA values are generally equilibrated and did not reveal significant fluctuations with average SASA values for iNOS-APO, iNOS-TAV, iNOS-ARG and iNOS-STD being 226.61 ± 4.94, 225.75 ± 4.57, 221.18 ± 4.03 and 229.86 ± 4.80 nm^2^, respectively. Consistent SASA values (≈225 nm^2^) indicated that the ligand–protein interface was solvent-shielded, with a hydrophobic core remaining intact.

#### 2.2.4. Intra and Inter Hydrogen Bond

**Figure 5:**
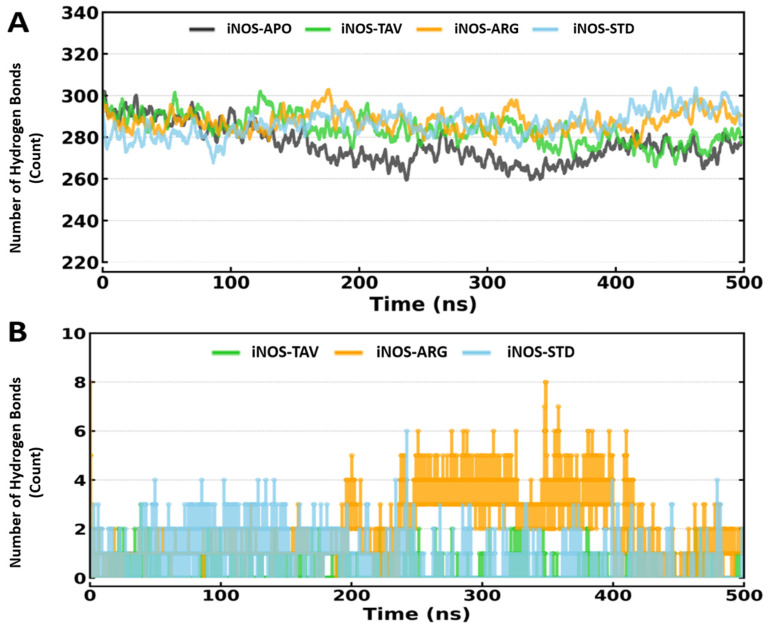
(**A**) Intramolecular hydrogen bonds of iNOS-APO, iNOS-TAV, iNOS-ARG, and iNOS-STD. (**B**) Intermolecular hydrogen bonds of iNOS-TAV, iNOS-ARG, and iNOS-STD.

To evaluate the interactions of Protein alone (iNOS-APO, iNOS-TAV, iNOS-ARG, and iNOS-STD), we examined intra and inter Hydrogen Bond formation and plotted intra hydrogen bonds for iNOS-APO, iNOS-TAV, iNOS-ARG, and iNOS-STD complexes in a time-dependent manner (Figure 5A), with an average number of intramolecular hydrogen bonds of 276.29 ± 11.41, 283.68 ± 9.89, 287.58 ± 8.53 and 286.55 ± 9.48, respectively. The stability of the protein–ligand interactions can be assessed through hydrogen bonding. We explored how the number of hydrogen bonds between iNOS-TAV, iNOS-ARG, and iNOS-STD changed over time and plotted the results (Figure 5B). Throughout the simulation, all the docked complexes were stable with a minimum of 0 to 8 hydrogen bonds with iNOS-TAV, iNOS-ARG, and iNOS-STD. The dynamic hydrogen-bonding pattern (0–8 bonds throughout the trajectory) suggests a stable binding configuration of tavaborole that is compatible with functional modulation.

In addition to intermolecular hydrogen bonding, analysis of intramolecular hydrogen bonds revealed that ligand binding influenced the internal hydrogen-bonding network of iNOS. The iNOS-TAV complex showed a slightly reduced but more stable intramolecular hydrogen-bond count compared to the substrate-bound (iNOS-ARG) and standard inhibitor (iNOS-STD) complexes, indicating preservation of the protein’s internal hydrogen-bonding network. Although L-arginine formed a higher number of intermolecular hydrogen bonds with iNOS, these interactions exhibited greater temporal fluctuations across the simulation trajectory. In contrast, the iNOS–tavaborole complex displayed fewer but more consistently maintained intermolecular hydrogen bonds over time, suggesting a stable binding mode despite a lower absolute hydrogen-bond count.

#### 2.2.5. Principal Component Analysis (PCA)

**Figure 6:**
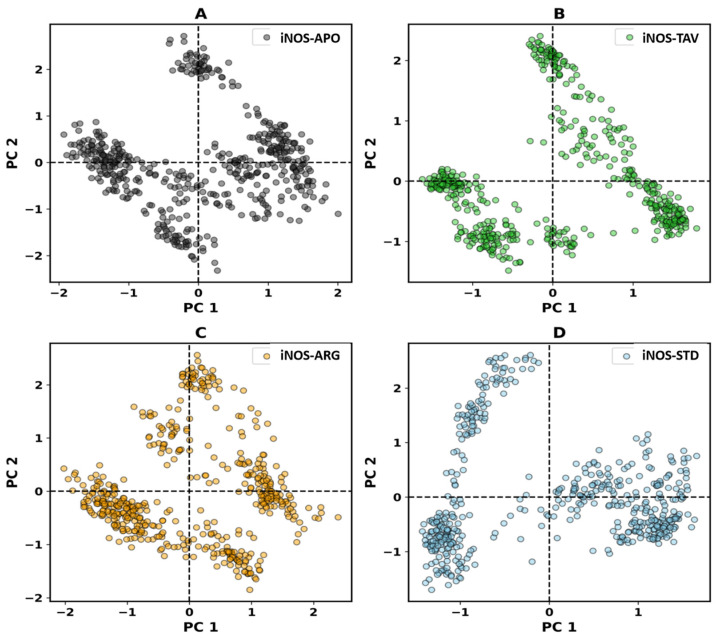
Two-dimensional projection PCA plot depicting the conformation sampling of (**A**) iNOS-APO, (**B**) iNOS-TAV, (**C**) iNOS-ARG, and (**D**) iNOS-STD on PC1 and PC2.

In PCA, the time evolution (indicates the global flexibility) of iNOS-APO, iNOS-TAV, iNOS-ARG, and iNOS-STD on both EVs was decreased, which implies stability (Figure 6). The plot clearly represents that iNOS-APO, iNOS-TAV, iNOS-ARG, and iNOS-STD covered nearly all the motions and overlapped. Overall, the fewer movements in the iNOS-APO, iNOS-TAV, iNOS-ARG, and iNOS-STD suggest that iNOS-APO, iNOS-TAV, iNOS-ARG, and iNOS-STD did not impact the dynamics and target conformation significantly, so this support the complex stability.

#### 2.2.6. Free Energy Landscapes (FELs)

**Figure 7:**
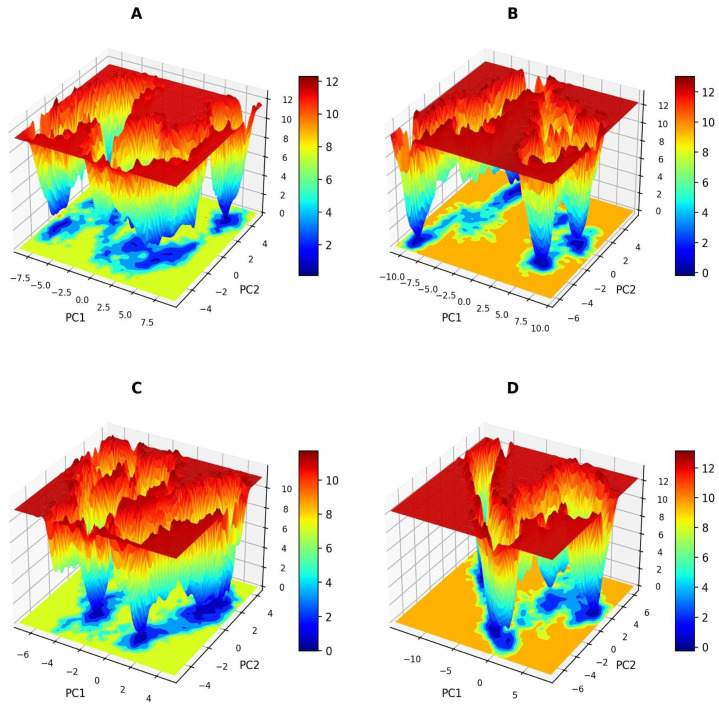
The free energy landscape plots for (**A**) iNOS-APO, (**B**) iNOS-TAV, (**C**) iNOS-ARG, and (**D**) iNOS-STD.

In the FEL plots for PC1 and PC2, the stable conformation of protein is represented by deeper blue colored regions. The energy values throughout the simulation of iNOS-APO, iNOS-TAV, iNOS-ARG, and iNOS-STD are 0 to 16 kJ/mol, and the FEL plots indicate that iNOS-APO, iNOS-TAV, iNOS-ARG, and iNOS-STD have only a single global minimum in a large local basin, which shows that the complex does not induce large-scale structural rearrangements in the target and stabilizes it (Figure 7). There was only one dominant energy basin, suggesting that the ligand was driven toward energetically favorable conformations.

#### 2.2.7. MM-PBSA

**Figure 8:**
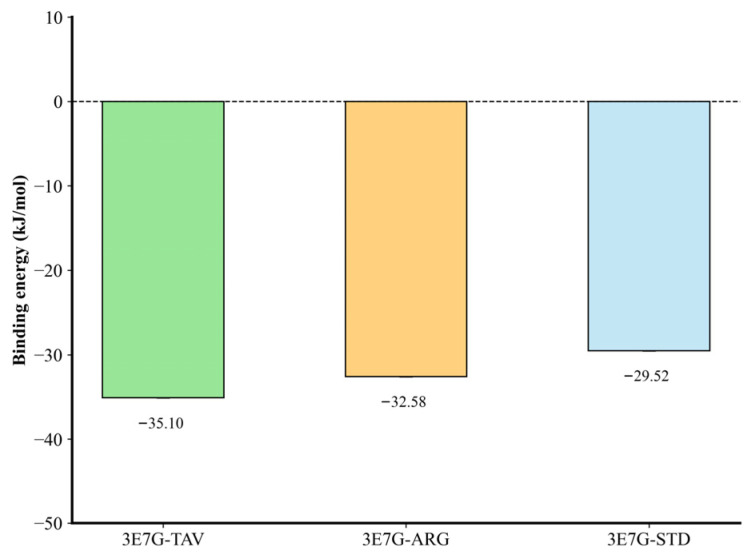
Focused energy contributions of key interacting residues (hotspot analysis) for the iNOS-TAV, iNOS-ARG, and iNOS-STD complexes derived from MM-PBSA calculations. These values represent relative residue-level interaction trends and do not correspond to the total MM-PBSA binding free energies reported in Table 2.

To investigate the binding affinity of iNOS-TAV, iNOS-ARG, and iNOS-STD, we compared their binding strengths using MM-PBSA analysis (through solvation, van der Waals, and electrostatic energies) and calculated the total binding free energy (kJ/mol) from an average of the last 50 ns of MD trajectories, to obtain quantitative information about interaction energetics (Table 2). The per-residue energy decomposition obtained from MM-PBSA analysis is shown in Figure 8, illustrating the relative residue-level interaction contributions (hotspot profiles) for the iNOS-TAV, iNOS-ARG, and iNOS-STD complexes. 

The iNOS-STD complex had the lowest binding free energy (–31.674 ± 25.960 kJ/mol), followed by the iNOS-TAV complex (18.382 ± 63.237 kJ/mol) and the iNOS-ARG complex (29.841 ± 13.121 kJ/mol), which suggests that Tavaborole forms a moderately stable association with the iNOS catalytic pocket. It is noted that the absolute binding free energies (ΔG_bind) calculated for the complexes exhibit positive values (Table 2). This is a well-documented limitation of the MM-PBSA method when applied to highly polar or charged active sites like the iNOS heme pocket. The continuum solvent model often overestimates the polar desolvation penalty (ΔG_polar) when using a standard solute dielectric constant (ε_in = 2). While increasing the dielectric constant (e.g., to ε_in = 4) is known to reduce this penalty and shift values toward negativity, we maintained the standard parameters to ensure rigorous methodological consistency with the reference inhibitor 1400W. Consequently, these values should be interpreted as relative ranking scores rather than absolute thermodynamic quantities. The stability of the complexes is independently confirmed by the structural metrics (RMSD, Rg, and Hydrogen Bonding) discussed previously. The observed variance in iNOS–tavaborole MM-PBSA binding free energy (±63.237 kJ/mol) predominantly originates from fluctuations in the polar solvation energy term (56.470 ± 54.146 kJ/mol), a known limitation of MM-PBSA calculations for small, partially polar ligands undergoing dynamic solvation-desolvation during binding. Despite this energetic heterogeneity, multiple independent stability descriptors confirm complex stability: RMSD (0.44 ± 0.10 nm), RMSF at catalytic site (<0.2 nm), radius of gyration (2.34 ± 0.02 nm), SASA (225.75 ± 4.57 nm^2^), hydrogen-bond persistence (0–8 bonds), PCA (restricted motion), and FEL (single energy basin). The relative binding trend (STD > TAV > ARG) remains robust, supported by per-residue energy decomposition. This interaction is mainly due to the favorable electrostatic and van der Waals contributions, which offset partially the unfavorable polar solvation energy, allowing for a stable of the ligand occupancy in an enzyme active site.

To further investigate the atomistic basis of binding, we performed per-residue MM-PBSA decomposition for the iNOS-TAV, iNOS-ARG, and iNOS-STD complexes (Table 3). Per-residue energy decomposition revealed distinct binding signatures among the three systems. While the total per-residue contribution for the iNOS-tavaborole complex (−6.47 kJ/mol) was weaker than that of the standard inhibitor 1400W (−20.47 kJ/mol), it was notably more favorable than the substrate-bound iNOS-arginine complex (7.83 kJ/mol). In the tavaborole complex, the dominant stabilizing contribution arose from the ligand itself, whereas individual active-site residues contributed modest but cooperative interactions. In contrast, L-arginine exhibited strong residue-level electrostatic contributions that were offset by unfavorable solvation effects, consistent with its weaker net binding affinity. Within the iNOS-TAV complex, residues TRP-372, TYR-373, and GLU-377 were identified as key interaction hotspots, providing van der Waals and electrostatic stabilization consistent with hydrogen bonding and π-π stacking interactions observed during MD simulations. This analysis provides mechanistic insight into how tavaborole engages the iNOS catalytic pocket at the atomistic level.

Notably, an interaction energy profile of iNOS-TAV mirrors the nonpolar and electrostatic distribution found in known iNOS-targeting inhibitors, which interact with conserved catalytic residues within the Rossmann fold superfamily, with which iNOS and arginase-1 share structural elements (in their catalytic or cofactor-binding domains) reflecting an evolutionarily conserved architecture of L-arginine-metabolizing enzymes, thus providing a structural basis for its dual compatibility in these enzymatic pockets, and suggesting that it may adopt a binding mode similar to known iNOS inhibitors, but with structural flexibility consistent with arginase-1.

## 3. Discussion

The inducible isoform (iNOS) is overexpressed in chronic inflammatory settings such as venous leg ulcers (VLUs), where sustained NO and peroxynitrite (ONOO^−^) production causes endothelial injury, oxidative–nitrosative stress, and delayed wound healing [15]. The current computational study shows that the approved antifungal agent tavaborole can bind within the iNOS catalytic pocket in a favorable manner and therefore represents a hypothesis-generating scaffold for potential repurposing in venous leg ulcers, pending experimental validation. Docking simulations showed that tavaborole had a binding affinity of −6.1 kcal/mol, similar to the L-arginine (substrate) (−6.0 kcal/mol) and 1400W (standard inhibitor) (−6.8 kcal/mol), and showed critical interactions with residues CYS200, ALA351, PHE369, GLY371, TRP372, TYR373, and GLU377 that are involved in iNOS catalysis and substrate stabilization [10,20]. The primary standard was 1400W due to its high selectivity, established crystallographic pose in the iNOS active site, and proven utility in computational validation studies, while multiple standards would have exponentially increased computational demands without additional mechanistic insight. Despite the close docking energies, L-arginine functions as a catalytic substrate characterized by transient, highly solvated interactions and substantial desolvation penalties, whereas tavaborole exhibits persistent π-π stacking, hydrophobic contacts, and stable residence within the catalytic pocket during long-timescale MD simulations, supporting its inhibitory potential beyond docking score magnitude alone. Tavaborole’s benzoxaborole scaffold contains a boron atom capable of reversible electrophilic interactions with nucleophilic residues or activated water molecules—a mechanism well-established in boron-based enzyme inhibitors. iNOS, as a redox-regulated, heme-containing enzyme with conserved catalytic residues (GLU371, TRP372, TYR373) and solvent-mediated chemistry, represents a plausible target for such interactions, extending beyond its known leucyl-tRNA synthetase inhibition [22,23,24].

To further validate the structural stability of the iNOS-tavaborole complex, 500 ns molecular dynamics simulations revealed early equilibration (~50 ns) and absence of substantial conformational drift; the iNOS-TAV complex showed an average RMSD of 0.44 ± 0.10 nm and Rg of 2.34 ± 0.02 nm—values well within the accepted range (0.4–0.5 nm) for large, multi-domain enzymes like iNOS over long simulations, with RMSD plateauing early and remaining stable thereafter. Taken together, these data indicate that tavaborole binding does not induce a significant conformational drift in iNOS [30,31]. Complementary metrics—RMSF at the catalytic site (<0.2 nm), SASA, PCA (restricted motion), and FEL (single energy basin)—collectively confirm preserved global compactness and absence of large-scale unfolding. The minimal variation in radius of gyration further supports this observation, confirming that tavaborole binding preserves the global compactness of iNOS and does not trigger large-scale unfolding or domain rearrangement. The number of observed energy minima (0–16 kJ/mol) and overlapping conformational sampling between the apo and complexed states also suggest a stable binding configuration [19,20]. Analysis of hydrogen-bonding dynamics suggested an inverse correlation between the number of intermolecular contacts and the net binding affinity. Although the natural substrate L-arginine formed a higher average number of intermolecular hydrogen bonds with iNOS compared to tavaborole, it exhibited a less favorable total binding free energy in MM-PBSA calculations. This apparent discrepancy can be attributed to the desolvation penalty; L-arginine is a highly polar, charged molecule that requires substantial energetic cost to shed its hydration shell before active-site engagement, reflected in its high positive polar solvation energy. In contrast, tavaborole, with a more balanced lipophilic profile, incurs a lower desolvation cost and achieves stable binding despite forming fewer, but more persistent, hydrogen bonds. Furthermore, analysis of intramolecular hydrogen bonds revealed that the iNOS-tavaborole complex maintained internal hydrogen-bonding patterns comparable to the apo protein, indicating preserved structural coherence, whereas the substrate-bound complex displayed greater fluctuations consistent with catalytic turnover. PCA and FEL mapping showed that global motion was restricted [32,33]. The localized increase in flexibility observed in peripheral loop regions of the iNOS-tavaborole complex reflects adaptive conformational responses to ligand binding and does not compromise catalytic-site integrity, a behavior commonly reported for stable enzyme-ligand complexes involved in chronic inflammatory and wound-healing pathways. These dynamic signatures suggest an optimal balance of inhibitory potential, in which iNOS functionality can be suppressed without destabilizing the structure and the consequent off-target toxicity that results from strong covalent inhibitors. The MM-PBSA calculations (binding free energy) validated the docking predictions with a total binding energy values of 18.38 ± 63.24 kJ/mol for tavaborole, stable to the level of the L-arginine (29.84 ± 13.12 kJ/mol) and 1400 W (−31.67 ± 25.96 kJ/mol) complexes, with nonpolar and polar components (−24.19 and −10.23 kJ/mol, respectively) that support favorable binding affinity, and a high polar solvation energy, as is common with small, hydrophilic molecules that would likely require further optimization of analogs in future development [34]. The MM-PBSA variance for tavaborole reflects characteristic polar solvation fluctuations of small-molecule inhibitors rather than binding instability. Comprehensive dynamic analyses and per-residue decomposition (TRP372, TYR373, GLU377 hotspots) confirm its energetically favorable interaction profile within the iNOS catalytic pocket, consistent with successful boron-based redox enzyme inhibitors. The hotspot residues identified in Table 3 correlate with hydrogen-bonding and π-π stacking interactions, confirming that TAV selectively targets key regions of the iNOS catalytic site, which may explain its dual compatibility with arginase-1.

From a mechanistic perspective, iNOS overexpression in VLUs contributes to vascular dysfunction, sustained inflammation, and impaired angiogenesis, which supports its relevance as a therapeutic target but does not by itself establish clinical efficacy [8,12,13,16]. The results of the current computational study suggest that reversible iNOS inhibition by tavaborole could, in principle, reduce nitrosative stress and facilitate endothelial recovery, but this remains a hypothesis that requires experimental confirmation. The dual regulation of the L-arginine metabolic axis—via iNOS suppression and arginase-1 modulation—has been proposed as an immunometabolic strategy for wound healing, but remains a conceptual framework that requires experimental validation [5,7,27]. Our previous work identified tavaborole as an arginase-1 inhibitor, and the current iNOS-focused study suggests that it can also engage the iNOS catalytic site; however, any dual in vivo modulation of arginase-1 and iNOS by tavaborole remains speculative and will require dedicated toxicity and pathway-selectivity studies before being considered a pharmacological outcome [25]. Notably, the physicochemical properties of tavaborole (low molecular weight, moderate lipophilicity, and skin permeability) support its topical formulations, and its safety profile in topical antifungal therapy supports its translational viability as a scaffold for future studies [22,23]. Tavaborole, which has a long safety record in topical antifungal therapy, is a translationally viable scaffold for chronic wound management compared to other iNOS inhibitors like 1400W and aminoguanidine that have difficulty in systemic administration. Additional mechanistic plausibility to this approach comes from recent computational and preclinical work exploring boron-based inhibitors for redox enzyme targeting [24]. In summary, the combined computational workflow (docking, MD simulation, and MM-PBSA energetics) provides a robust atomistic-level understanding of ligand-iNOS interactions that have been supported by the observed conformational stability, hydrogen bonding, and energetically favorable profiles with tavaborole as a low-toxicity candidate for iNOS modulation. In vitro enzyme inhibition assays, macrophage polarization studies, and topical efficacy in murine wound models will be necessary to validate the computational predictions. Future experimental validation will include in vitro iNOS inhibition assays, macrophage polarization studies (M1/M2 shift), and ex vivo human venous ulcer tissue models to confirm computational predictions.

## 4. Materials and Methods

### 4.1. Molecular Docking

In this study, molecular docking was carried out to evaluate the binding affinity of the ligands Tavaborole [TAV-test], L-Arginine [ARG-substrate], and 1400W [STD-standard] toward the human inducible nitric oxide synthase enzyme [PDB ID: 3E7G, resolution 2.20 Å]. The chemical structure of all three ligands and standard was downloaded from PubChem and subsequently converted to three-dimensional (3D) format using Open Babel 3.1.1 [35]. For the docking of tavaborole, the boron atom was treated as a non-hydrogen heavy atom. During the PDBQT conversion, Open Babel 3.1.1 assigns generic van der Waals parameters (equivalent to aromatic carbon) to the boron atom, which is a standard approximation for benzoxaborole scaffolds in AutoDock 4.2 given the lack of specific boron calibration in the default force field. The benzoxaborole ring was maintained as a rigid planar moiety during docking. No manual customization of boron parameters was applied during the docking protocol. Although treating boron as a carbon equivalent in AutoDock 4.2 is an approximation, it is a standard approach for benzoxaboroles and is considered sufficient for predicting binding poses. Therefore, the docking results served primarily as a starting point to select the best conformation for rigorous molecular dynamics simulations. The prepared ligands were docked with AutoDock 4.2 [36] to predict protein–ligand interactions. The docking grid was centered on the geometric centroid of the co-crystallized inhibitor and heme iron in the iNOS crystal structure (PDB ID: 3E7G), corresponding to the canonical L-arginine/BH_4_/heme catalytic pocket defined by key residues GLU371, TRP372, TYR373 (center_x = 55.486, center_y = 20.407, center_z = 78.597 Å; grid box 12 × 12 × 16 Å). These coordinates and catalytic residues are well-documented in iNOS structural and mutagenesis studies [20,36,37]. Tavaborole docking results demonstrated favorable binding affinity and interactions consistent with iNOS inhibition. We selected 1400W as the reference standard because it is a well-characterized, highly selective iNOS inhibitor with a known crystallographic binding mode in the iNOS active site (PDB 3E7G), making it an appropriate benchmark for docking and MD validation. Including additional standards (aminoguanidine, L-NIL) would substantially increase computational cost without proportionate mechanistic gain. The top-scoring binding poses were selected for visualization and analysis using PyMOL 2.6 to determine hydrogen bonding, hydrophobic, and π-π stacking key residues involved in the protein–ligand interactions. Molecular docking was performed in triplicate for each ligand, and the best-scoring pose from Trial 1 with the most favorable binding energy and optimal orientation within the catalytic pocket was chosen for the subsequent molecular dynamics simulations.

### 4.2. Molecular Dynamics

The dynamics were considered for the crystal structure of the protein–inhibitor complex. The complexes of iNOS–Protein Alone [iNOS-APO], iNOS–Tavaborole [iNOS-TAV], iNOS–L-arginine [iNOS-ARG], and iNOS–1400W [iNOS-STD] selected for docking analysis were used. The ligand topology and force field parameters for tavaborole were generated using the CHARMM General Force Field (CGenFF) program via the CHARMM-GUI Ligand Reader & Modeler. The boron atom was parameterized using the specific atom type ‘B3O2’ (or the specific type from your str file, likely associated with boronic esters), with partial charges and bonded parameters derived by analogy to existing boronate derivatives in the CGenFF database. Parameter penalty scores were evaluated to ensure the reliability of the force field assignment for the benzoxaborole core. We did not manually adjust the parameters for tavaborole. Instead, we used the bonded and non-bonded parameters, including the partial charges for boron, exactly as generated by the CHARMM-GUI/CGenFF interface. Addition of hydrogens to the heavy atoms were done using the GROMACS 2020.4-pdb2gmx module. Using steepest descent, vacuum minimization (1500 steps). The structures were then solvated in a In a cubic periodic box, the structures were solvated using the water model of simple point charge (SPC/E). Then, the appropriate amounts of Na and Cl counter ions were added to maintain a physiological salt concentration (0.15 M). Temperature coupling was performed using the Nose-Hoover thermostat (310 K, τ = 0.5 ps). Pressure coupling employed the Parrinello–Rahman barostat (1.0 bar, τ = 2.0 ps). All bond lengths were constrained using the LINCS algorithm (expansion order 4, 1.0 × 10^−5^ precision). The leap-frog integrator was used with a 2 fs time step. Based on a previous literature, the system preparation was done. All structures that resulted after the NPT equilibration stage were run for 500 ns under NPT ensemble conditions (equilibration ~50 ns, MM-PBSA calculated from final 50 ns). To facilitate full reproducibility of this work, we have provided a comprehensive Supplementary Data archive containing all AutoDock 4.2 preparation files, the CGenFF ligand topologies and parameters, and the complete GROMACS input configuration files for the iNOS-APO, iNOS-TAV, iNOS-ARG, and iNOS-STD systems.

Finally, several tools from the GROMACS 2020.4 software were employed for the trajectory analysis: hydrogen bonding (H-Bond), the protein root mean square deviation (RMSD), radius of gyration (RG), root mean square fluctuation (RMSF), and solvent accessible surface area (SASA). The molecular mechanics Poisson–Boltzmann surface area (MM-PBSA) approach was used to analyze the binding free energy (iNOS-TAV, iNOS-ARG, and iNOS-STD) of an inhibitor with protein over simulation time, which was estimated using the GROMACS utility g_mmpbsa [38]. We calculated iNOS-TAV, iNOS-ARG, and iNOS-STD for the last 50 ns with dt 1000 frames to ensure accuracy.

The collective motions in the protein complexes were explored using Principal Component Analysis (PCA). The simulation was analyzed for the first few EVs, corresponding to the largest modes of motion, and the time evolution of PCA was used to examine overall flexibility of the complexes, where lower overall flexibility on both EVs indicated stability in the complexes. To gain further insight into the general stability and protein folding techniques, Free Energy Landscapes (FELs) mapping provide the insights about general stability and protein folding with the visualization of the most stable conformational ensembles by energy level mapping across different states of conformations. FEL plots were generated for PC1 and PC2, and the regions in deeper blue with lower energy and higher stability that indicating favorable protein conformations [39,40].

## 5. Conclusions

This computational study demonstrates that tavaborole binds stably to the iNOS catalytic domain through molecular docking, 500 ns molecular dynamics simulations, and MM-PBSA analyses, forming favorable interactions with key residues (TRP372, TYR373, GLU377). These findings provide a mechanistic and computational basis for investigating tavaborole as a potential iNOS inhibitor, rather than direct therapeutic proof. Future experimental validation through in vitro iNOS inhibition assays, macrophage polarization studies, and ex vivo venous ulcer models is required to confirm its capacity to reduce nitrosative stress and support wound healing, leveraging its established topical safety profile.

## Figures and Tables

**Table 1:** Docking scores of ligands towards the target protein iNOS (PDB ID: 3E7G].

Ligands	Trial 1	Trial 2	Trial 3
Tavaborole (TAV, Test)	−6.1	−6.1	−6.1
L-arginine (ARG, Substrate)	−6.0	−5.9	−5.8
1400W (STD, Standard)	−6.8	−6.8	−6.8

**Table 2:** MMPBSA—energy summary of three docked complexes.

System	Van Der Waals Energy	Electrostatic Energy	Polar Solvation Energy	Binding Energy
iNOS-TAV	−24.189 +/− 21.316 kJ/mol	−10.236 +/− 11.362 kJ/mol	56.470 +/− 54.146 kJ/mol	18.382 +/− 63.237 kJ/mol
iNOS-ARG	−51.802 +/− 5.279 kJ/mol	−144.113 +/− 17.168 kJ/mol	234.668 +/− 12.907 kJ/mol	29.841 +/− 13.121 kJ/mol
iNOS-STD	−88.533 +/− 9.276 kJ/mol	−23.574 +/− 18.717 kJ/mol	92.429 +/− 34.715 kJ/mol	−31.674 +/− 25.960 kJ/mol

**Table 3:** Per-residue energy decomposition of iNOS-TAV, iNOS-ARG, and iNOS-STD complexes (MM-PBSA, kJ/mol).

Residue of iNOS	TAV	ARG	STD
GLN-263	−0.0619	1.5983	−0.1438
TYR-347	0.0206	−0.8131	−0.1201
PRO-350	0.0299	−3.1334	−1.341
ALA-351	−0.0073	0.1298	−0.3186
VAL-352	0.0171	−1.1815	−1.0512
PHE-369	0.0641	−0.8137	−2.9737
ASN-370	0.1044	0.1486	0.561
GLY-371	0.0291	0.302	−0.3977
TRP-372	0.153	−0.6454	−1.2628
TYR-373	−0.0012	−6.2619	−2.4361
GLU-377	0.0802	15.7413	17.0023
Ligand	−6.8944	2.7591	−27.9923
**Total**	−6.4664	7.8301	−20.474

## Data Availability

The original contributions presented in this study are included in the article/Supplementary Material. Further inquiries can be directed to the corresponding authors.

## Supplementary Materials

The following supporting information can be downloaded at: https://www.mdpi.com/article/10.3390/ph19010137/s1, Figure S1: Radius of gyration (Rg) analysis of iNOS-APO, iNOS-TAV, iNOS-ARG, and iNOS-STD complexes over the 500 ns simulation. Supplementary data S2: Compressed archive containing all docking input files and molecular dynamics input and output data, including AutoDock 4.2 ligand preparation files (.pdbqt), CHARMM-GUI/CGenFF ligand topology and parameter files for tavaborole, and complete GROMACS input files and trajectory data for the iNOS-APO, iNOS-TAV, iNOS-ARG, and iNOS-STD systems.

## Abbreviations

The following abbreviations are used in this manuscript:

ALA	Alanine
ARG	L-Arginine
ARG1	Arginase-1
APO	Apo form (unbound protein structure)
BH_4_	Tetrahydrobiopterin
CVI	Chronic Venous Insufficiency
CYS	Cysteine
ΔG_binding	Gibbs Free Energy of Binding
eNOS	Endothelial Nitric Oxide Synthase
EV	Eigenvalue
FEL	Free Energy Landscape
GLN	Glutamine
GLU	Glutamic Acid
GLY	Glycine
H-Bond	Hydrogen Bond
IL-1β	Interleukin-1 Beta
IL-6	Interleukin-6
iNOS	Inducible Nitric Oxide Synthase
kJ/mol	Kilojoules per mole
LPS	Lipopolysaccharide
MD	Molecular Dynamics
MM-PBSA	Molecular Mechanics Poisson–Boltzmann Surface Area
MMP	Matrix Metalloproteinase
M1/M2	Macrophage Phenotypes (Pro-inflammatory/Reparative)
NO	Nitric Oxide
ONOO^−^	Peroxynitrite
PCA	Principal Component Analysis
PDB	Protein Data Bank
PHE	Phenylalanine
PRO	Proline
Rg	Radius of Gyration
RMSD	Root Mean Square Deviation
RMSF	Root Mean Square Fluctuation
RNS	Reactive Nitrogen Species
ROS	Reactive Oxygen Species
SASA	Solvent-Accessible Surface Area
STD	Standard Inhibitor (1400W)
TNF-α	Tumor Necrosis Factor-Alpha
TRP	Tryptophan
TYR	Tyrosine
TAV	Tavaborole
VLU	Venous Leg Ulcer
