# Supplementary material for: Decoding iNOS Inhibition: A Computational Voyage of Tavaborole Toward Restoring Endothelial Homeostasis in Venous Leg Ulcers
Source: Pharmaceuticals (Basel). 2026 Jan 13;19(1):137. doi: 10.3390/ph19010137 (PMC12845252; doi:10.3390/ph19010137)
Supplement: Supplementary file 1 [file pharmaceuticals-19-00137-s001.zip › pharmaceuticals-4013697-supplementary.pdf]

Radius of gyration (Rg)

The calculation of Rg was done and plotted over time to examine compactness and dynamic stability of the iNOS-APO, iNOS-TAV, iNOS-ARG, and iNOS-STD complex (Figure 4), with an average Rg of 2.33 ± 0.02, 2.34 ± 0.02, 2.30 ± 0.02 and 2.36 ± 0.02 nm, respectively.

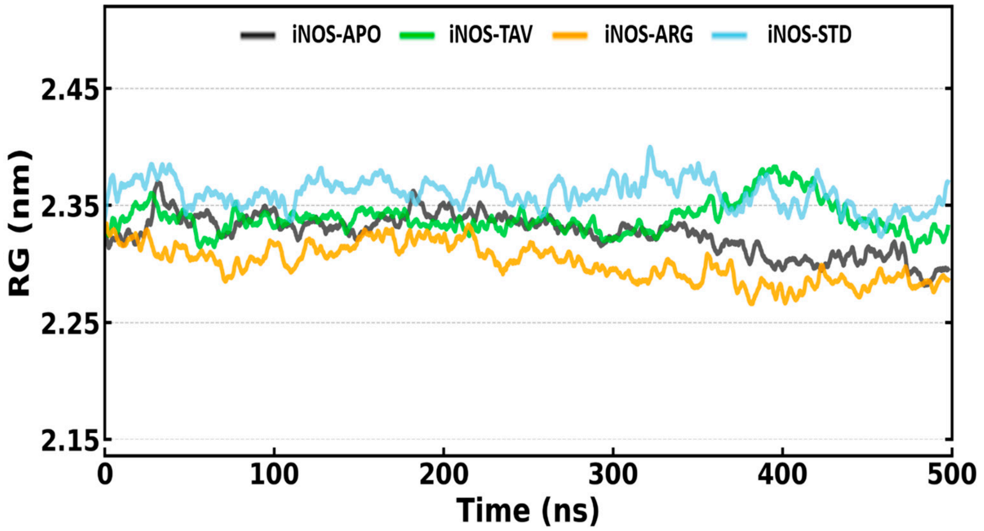

Figure S1. Radius of gyration (Rg) analysis of iNOS–APO, iNOS–TAV, iNOS–ARG, and iNOS–STD complexes over the 500 ns simulation.

Supplementary Data S2:
https://figshare.com/s/c3ef47373cf33bbb2d00
